# The common origin and degenerative evolution of flagella in *Actinobacteria*

**DOI:** 10.1128/mbio.02526-23

**Published:** 2023-11-29

**Authors:** Siqi Zhu, Xian Sun, Yuqian Li, Xueyin Feng, Beile Gao

**Affiliations:** 1 CAS Key Laboratory of Tropical Marine Bio Resources and Ecology, Guangdong Key Laboratory of Marine Materia Medica, Innovation Academy of South China Sea Ecology and Environmental Engineering, Guangdong Provincial Observation and Research Station for Coastal Upwelling Ecosystem, South China Sea Institute of Oceanology, Chinese Academy of Sciences, Guangzhou, Guangdong, China; 2 Tropical Marine Biological Research Station in Hainan, Chinese Academy of Sciences and Hainan Key Laboratory of Tropical Marine Biotechnology, Sanya, Hainan, China; 3 Southern Marine Science and Engineering Guangdong Laboratory (Guangzhou), Guangzhou, Guangdong, China; 4 University of Chinese Academy of Sciences, Beijing, China; The Ohio State University, Columbus, Ohio, USA

**Keywords:** *Actinobacteria*, flagella, chemotaxis, c-di-GMP, evolution

## Abstract

Species of the phylum *Actinobacteria* have long time been thought mostly non-motile and non-flagellated, in spite of their extraordinary diversity in morphology, physiology, and ecology. It remains unclear how very few actinobacterial species acquired their flagellar genes and the role of flagellar motility in the evolution of different lineages within this large phylum. Here, we performed a comprehensive phylogenomic analysis of flagellar components in all actinobacterial species, including the deepest branches of this phylum identified recently. Our results provide robust evidence that all actinobacterial flagella shared a common origin and likely evolved from their last common flagellated ancestor by vertical inheritance. Later lineages underwent massive flagellar losses with the ecological switch from aquatic to terrestrial land or host-associated environments. In addition, the actinobacterial flagella also showed degenerative evolution in terms of their composition, leading to the simplest rod known so far. Furthermore, these evolutionary changes of flagella were accompanied by noteworthy variations in the chemosensory system and c-di-GMP mediated signaling network. Overall, we present here a detailed picture of flagellar evolution in *Actinobacteria*, which provides new insights into the evolution of major actinobacterial lineages and previously unobserved links between flagella and other biological features. The simplest flagellar rod as a product of degenerative evolution could possibly serve as a model for future nanomachine reconstruction in synthetic biology.

**IMPORTANCE:**

Flagellar motility plays an important role in the environmental adaptation of bacteria and is found in more than 50% of known bacterial species. However, this important characteristic is sparsely distributed within members of the phylum *Actinobacteria*, which constitutes one of the largest bacterial groups. It is unclear why this important fitness organelle is absent in most actinobacterial species and the origin of flagellar genes in other species. Here, we present detailed analyses of the evolution of flagellar genes in *Actinobacteria*, in conjunction with the ecological distribution and cell biological features of major actinobacterial lineages, and the co-evolution of signal transduction systems. The results presented in addition to clarifying the puzzle of sporadic distribution of flagellar motility in *Actinobacteria*, also provide important insights into the evolution of major lineages within this phylum.

## INTRODUCTION

The bacterial flagellum is one of the most complex nanomachines that is made of dozens of different protein components (1, 2). It is anchored in the cell envelope and mostly with extracellular filaments longer than the bacterial cell body. The presence or absence of flagellar structure in a species has a direct effect on its cell biology including morphology, physiology, and development (3, 4). Meanwhile, flagella-mediated motility plays an important role in bacterial environmental adaptation since motile or non-motile lifestyle drastically changes the nutrient uptake and interaction with other organisms (5, 6). For example, the endoflagella in *Spirochetes* are central for their singularly spiral shape and pathogenesis within hosts (7). In *Caulobacter crescentus* with complex cell cycles, flagellar assembly is strictly coordinated with cell cycle progression and cell differentiation (8, 9). Compared to marine copiotrophs, marine oligotrophs with streamlined genomes are generally non-motile and the absence of chemotaxis and motility genes reflects their strategy to live off low nutrient concentrations (10). Therefore, it is likely that flagella and flagella-mediated motility have played important roles in bacterial evolution, such as speciation (11–13). However, the role of flagellar gain or loss in “ancient” speciation events that lead to lineage divergences has not been systematically investigated at higher taxonomic levels such as phylum, class, or order.

The phylum *Actinobacteria* comprising Gram-positive bacteria, constitutes one of the largest phyla in the *Bacteria* domain, but very few species from this phylum have been studied for their flagella (14, 15). The actinobacterial species show great variety in ecology, morphology, physiology, and life cycles, a diverse bacterial phylum extraordinaire (16, 17). Most studied species of this phylum are those with important medical and industrial values, including the top human pathogens from the genus *Mycobacterium*, foremost natural product producers from the *Streptomyces* and related genera, probiotic agents from the genus *Bifidobacterium* (18). All of these species that are regarded as representatives of *Actinobacteria* happen to lack flagella and are non-motile. Earlier studies suggest that very few actinobacterial species have flagella, and surprisingly some species can form flagellated spores for a short period during their lifecycles, so-called “zoospores” that swim at very high speed (135–160 µm/s for zoospores in comparison with 25 µm/s for *Escherichia coli*) (19–22). However, these were only electron microscopic observations of flagellar filaments or motility tests, and functional studies on flagella of actinobacterial species are very scarce. To our knowledge, the only actinobacterial species whose flagella were genetically characterized is *Actinoplanes missouriensis* with zoospores (23, 24). Compared to other bacterial phyla, little to nothing is known about what the actinobacterial flagellar motors look like, how they rotate through the thick peptidoglycan layer or zoospore envelopes, and the relationship of flagellar motility and the extraordinary biological diversity of actinobacterial species.

A seminal study in 2008 investigated the flagellar gene distribution in 516 bacterial genomes including 43 from the phylum *Actinobacteria* (25). Mostly based on the knowledge gained from the flagellum of Gram-negative model *Salmonella enterica* serovar Typhimurium (*S*. Typhimurium hereafter), this study indicated that only four actinobacterial genomes encode “incomplete” flagellar gene set and flagellar genes are entirely absent in the rest actinobacterial genomes (25). Importantly, the authors raised a question that still remains elusive: how have actinobacterial species acquired their flagellar genes? Are they derived from a motile ancestor with flagellar loss in most lineages, or by horizontal gene transfer (HGT)? With the availability of genome sequences for ~500 actinobacterial species providing extensive coverage of genetic diversity within *Actinobacteria*, it is necessary to re-examine the distribution of flagellated species within this phylum, in conjunction with the updated knowledge of the flagellar machinery and the biological context of different species/lineages. Such studies could provide important insights concerning the origin/distribution of flagellated species within this phylum and the functional role of this important machinery in the properties and diversification of species from this vast and diverse phylum.

Here, we systematically analyzed the phylogenetic distribution of flagellar genes in the phylum *Actinobacteria*, covering all known species with complete genomes and species with nearly complete genomes from basal branches, particularly three novel classes (*Ca*. Aquicultoria, *Ca*. Geothermincolia, and *Ca*. Humimicrobiia) (26). In addition, to integrate the flagellar evolution within ecological and evolutionary (eco–evo) context, we have collected information regarding the isolation niches, cell biology, and lifestyle characteristics of each species by literature search. Finally, we have examined the co-evolution of flagella and two related signal transduction systems including chemosensory and c-di-GMP mediated systems, in order to present a more comprehensive picture of flagellar evolution. Our results provide important insights regarding the origin and evolution of flagella in *Actinobacteria* and the evolution of the bewildering diversity of species in this phylum.

## RESULTS

### Highly biased flagellar distribution among aquatic unicellular classes and terrestrial filamentous *Actinomycetia*

**Fig 1:**
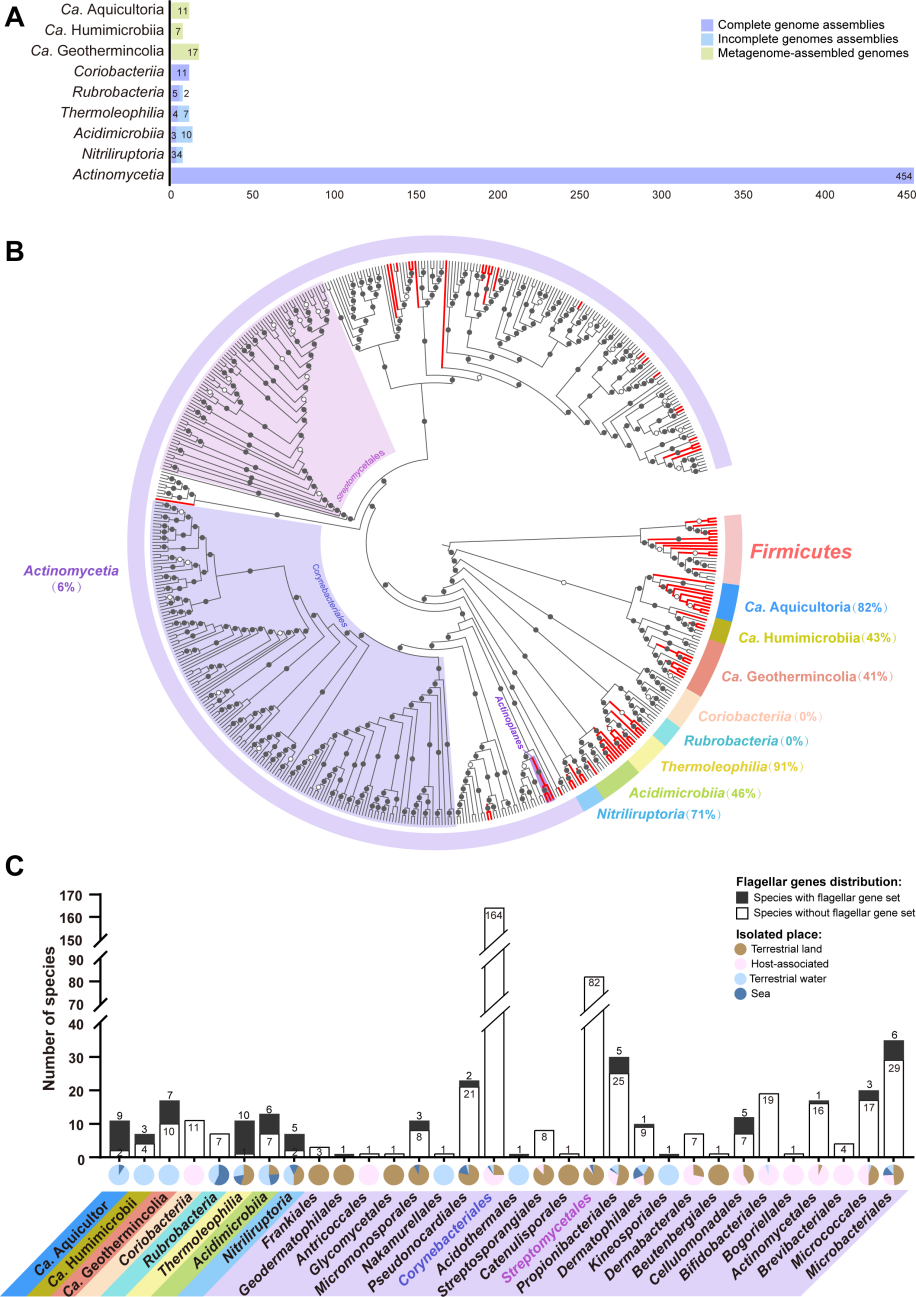
Highly biased distribution of flagellated species across *Actinobacteria*. (**A**) Analyzed genomes of each class from the phylum *Actinobacteria*. (**B**) The distribution of flagellated species on the species tree of *Actinobacteria*. Circles above each node within the tree represent bootstrap values (*n* = 1,000 replicates) of ≥90% (solid) or ≥70% (hollow). Branches representing flagellated species are highlighted in red and the ratios of species with flagellar genes for each class are shown in brackets. The details of this tree regarding species name supplemented with ecological and genomic features are shown in Fig. S1. (**C**) The proportions of flagellated species (column) and ecological distribution (pie chart) are displayed for eight classes, while the largest class *Actinomycetia* shown at the order level.

The phylum *Actinobacteria* currently includes six defined classes with the class *Actinomycetia* being the largest, accounting for more than 95% of isolated species in this phylum (Fig. 1A) (14, 15). To ensure the greatest species diversity, we compiled a set of 480 completely sequenced actinobacterial genomes, with only one genome selected for the same species. In addition, we added 23 nearly complete genome assemblies from the underrepresented four classes other than *Actinomycetia*, and 35 metagenome-assembled genomes (MAGs) with high quality from three recently identified actinobacterial classes (Fig. 1A) (26). Totally 538 genomes from all known nine classes were analyzed for this study, thus far constituting a relatively comprehensive representative species database for *Actinobacteria* (Table S1).

Overall, we identified 69 genomes with a flagellar gene set, suggesting that only 13% of actinobacterial species are flagellated from our genomic repository (Table S2; Fig. S1). This ratio is much less than the average 50% flagellated species in the *Bacteria* domain (25), corroborating the previous impression that very few actinobacterial species have flagella (20, 25). However, when these flagellated species were mapped in a robust phylogenetic tree for all analyzed actinobacterial species, a highly biased distribution was seen among different classes (Fig. 1B). The basal branches including *Ca*. Aquicultoria, *Ca*. Geothermincolia, *Ca*. Humimicrobiia, *Thermoleophilia*, *Acidimicrobiia*, and *Nitriliruptoria* have 41%–91% species with flagellar gene clusters, similar to other bacterial phyla that are densely populated with flagellated species. In contrast, only 6% of species in the biggest class *Actinomycetia* have flagellar genes, and 0 in the two classes *Coriobacteriia* and *Rubrobacteria* (Fig. 1B; Fig. S1).

Given the strong bias of flagellar distribution among actinobacterial classes, we were curious to know whether it is related to specific ecosystems, lifestyle, or cell biology. Among the basal classes that have more than 40% species with flagellar genes, their isolation places are mostly aquatic environments, such as sea, hot spring, or groundwater (Fig. 1C; Table S1). In addition, species of these basal classes are all unicellular non-spore-forming bacteria, different from the class *Actinomycetia* with great diversity. However, none of the *Rubrobacteria* species analyzed here have flagellar gene sets, in spite of the fact that they are also aquatic unicellular bacteria (Fig. 1C; Table S1). Notably, all known species from *Rubrobacteria* currently belong to the genus *Rubrobacter*, so whether a single genus can represent this class awaits confirmation with more isolates. Unlike the above free-living basal classes, species of *Coriobacteriia* are all isolated from humans or animals, suggesting that a strictly host-associated lifestyle might be related to the flagellar loss in this class (Fig. 1C; Table S1).

The sparsely flagellated *Actinomycetia* have a majority of species dwelling in soil (46%) or associated with hosts (43%), and only 11% either in sea or terrestrial water (Fig. 1C; Table S1). To dissect the species of this large class, we examined their ecological and biological characteristics at the taxonomic level of order. The largest order without any flagellated species is *Corynebacteriales,* with 65% host-associated species (Fig. 1C; Table S1). In addition to host association, species of this order have unique cell envelope structures including a layer of arabinogalactan and an outer membrane made of mycolic acid, even more complex than the typical outer membrane of Gram-negative bacteria (27, 28). Thus, it is tempting to speculate that if a flagellar rod is anchored in this arabinogalactan-mycolic acid arrangement, its composition and structure will be different from the classical Gram-negative bacteria. On the other hand, the absence of flagella in all identified *Corynebacteriales* species might be related to the appearance of an unusual outer membrane structure, which we think is a previously overlooked link. The second largest order without flagellar genes is soil-dwelling *Streptomycetales*, whose hallmark features are complex morphology and life cycles including substrate mycelium, aerial mycelium, and exospore formation (Fig. 1C; Table S1) (29). The filamentous growth of mycelium is the opposite of free-moving single cell, which might be the reason why species of *Streptomycetales* and related orders such as *Streptosporangiales* within *Actinomycetia* do not have flagella.

Taken together, our investigation of flagellar gene distribution in actinobacterial genomes suggests that aquatic unicellular species tend to have flagella, while terrestrial filamentous species or host-associated species generally don’t. However, some species of *Actinomycetia* can form flagellated zoospores in a very short period after mycelium growth during their life cycle (19, 24), which will be further discussed about their unusual signaling network later.

### The common origin of actinobacterial flagella illuminates a motile ancestor

**Fig 2:**
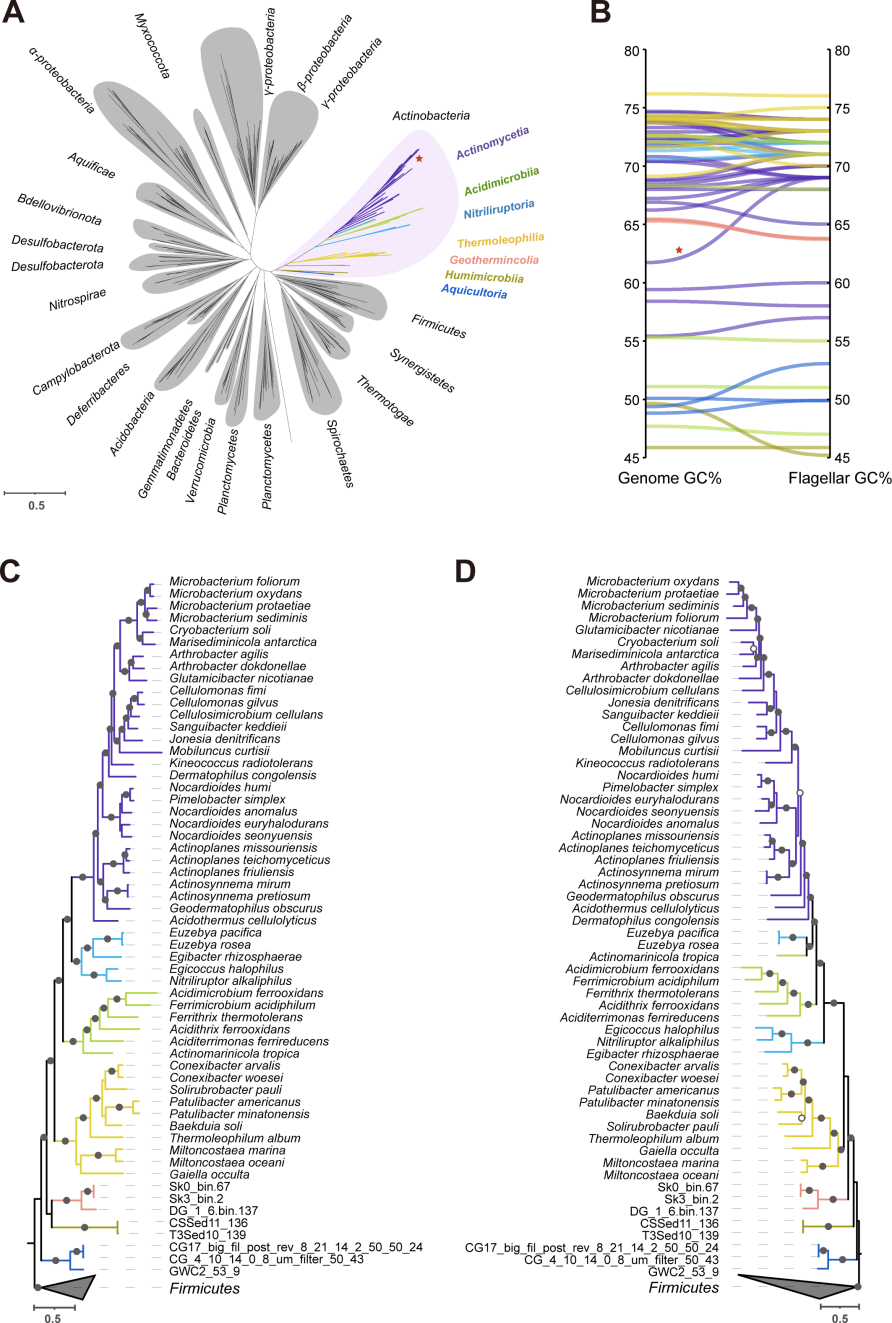
The common origin and vertical inheritance of flagellar genes in *Actinobacteria*. (**A**) An unrooted phylogenetic tree based on 12 concatenated flagellar proteins from diverse bacterial phyla. The branch colors of *Actinobacteria* are according to different classes. (**B**) Comparison of GC content between the whole genome sequence and the flagellar gene cluster for each flagellated species. The only species with GC% difference >5% is marked with a red star in both panels **A** and **B**. (**C**) Species tree based on 120 concatenated marker proteins from flagellated species in *Actinobacteria*, rooted with *Firmicutes*. (**D**) Flagellar tree based on 12 concatenated flagellar proteins from flagellated species in *Actinobacteria*, rooted with *Firmicutes*. Circles above each node in both (**C**) and (**D**) represent bootstrap values (*n* = 1,000 replicates) of ≥90% (solid) or ≥70% (hollow).

To approach the unresolved question how actinobacterial species acquired flagella, we constructed a phylogenetic tree based on concatenated flagellar core proteins from all flagellated actinobacterial species and 299 flagellated species of 17 different bacterial phyla, covering all major bacterial lineages (Fig. 2A). Twelve flagellar core proteins including FlhA, FlhB, FliP, FliQ, FliR, FliI, FliF, FliG, FiM, FliE, FlgB, and FlgC were used for phylogenetic analysis, which are universally present, highly conserved, and encoded by single-copy gene. In this flagellar protein tree, all actinobacterial species form a single cluster, clearly separate from the other bacterial phyla. In addition, all the flagellar gene clusters in actinobacterial genomes have relatively conserved gene order arrangement, which is likely a result of vertical inheritance or HGTs only within this phylum (Fig. S2). Furthermore, we compared the GC content of the flagellar gene cluster and the whole genome sequence for each flagellated actinobacterial species. Almost all species have identical or very similar GC content for their flagellar genes and the rest of the genome, except that only one genome displayed more than a 5% difference but its flagellar proteins robustly grouped with other actinobacterial homologs in the tree (Fig. 2A and B). Altogether, these results strongly suggest that flagellar core proteins from actinobacterial species share a common origin and there is no inter-phyla HGT between *Actinobacteria* and others.

To further examine the evolutionary path of flagellar genes within *Actinobacteria*, we compared the branching pattern of the species tree and flagellar protein tree of flagellated species (Fig. 2C and D). Overall, these two trees showed very similar topology, with three novel classes as the deepest branch, followed by *Thermoleophilia*, and the class *Actinomycetia* being the latest. There is only one inter-class exchange of flagellar proteins between *Acidimicrobiia* and *Nitriliruptoria* (Fig. 2C and D). These phylogenetic analyses suggest that flagellar evolution followed species evolution in the phylum *Actinobacteria* except very few HGTs within this phylum. Based on the flagellated species distribution in the phylogenetic framework of the whole phylum (Fig. 1B) and the common origin of actinobacterial flagellar core proteins (Fig. 2A), it is most likely that the last common ancestor of *Actinobacteria* already had flagella and vertically passed on to its descendants. Furthermore, although the *Actinomycetia* species are rarely flagellated, their flagellar proteins evolved from the actinobacterial ancestor and many of its lineages lost flagellar genes, likely due to adaptation to filamentous growth in soil or parasitism with hosts (Fig. 1B).

Therefore, the distribution of flagellar genes in actinobacterial species that we see today is a result of a combination of vertical inheritance from a motile ancestor and massive gene loss in later lineages.

### The degenerative evolution of flagella in *Actinobacteria* leads to a simpler rod

The absence of any information on the structure and mechanisms of actinobacterial flagellar motors promoted us to harness genomic evidence to predict how the motors would be in actinobacteria. Notably, almost all flagellar genes are encoded in one big cluster in actinobacterial genomes, with very few cases of 1–2 genes located apart from the flagellar gene cluster (Fig. S3). The highly clustered nature of flagellar genes in actinobacterial genomes suggests that it is less likely for unknown flagellar structural components (not including unknown regulatory genes) to be missed from our genomic analyses.

**Fig 3:**
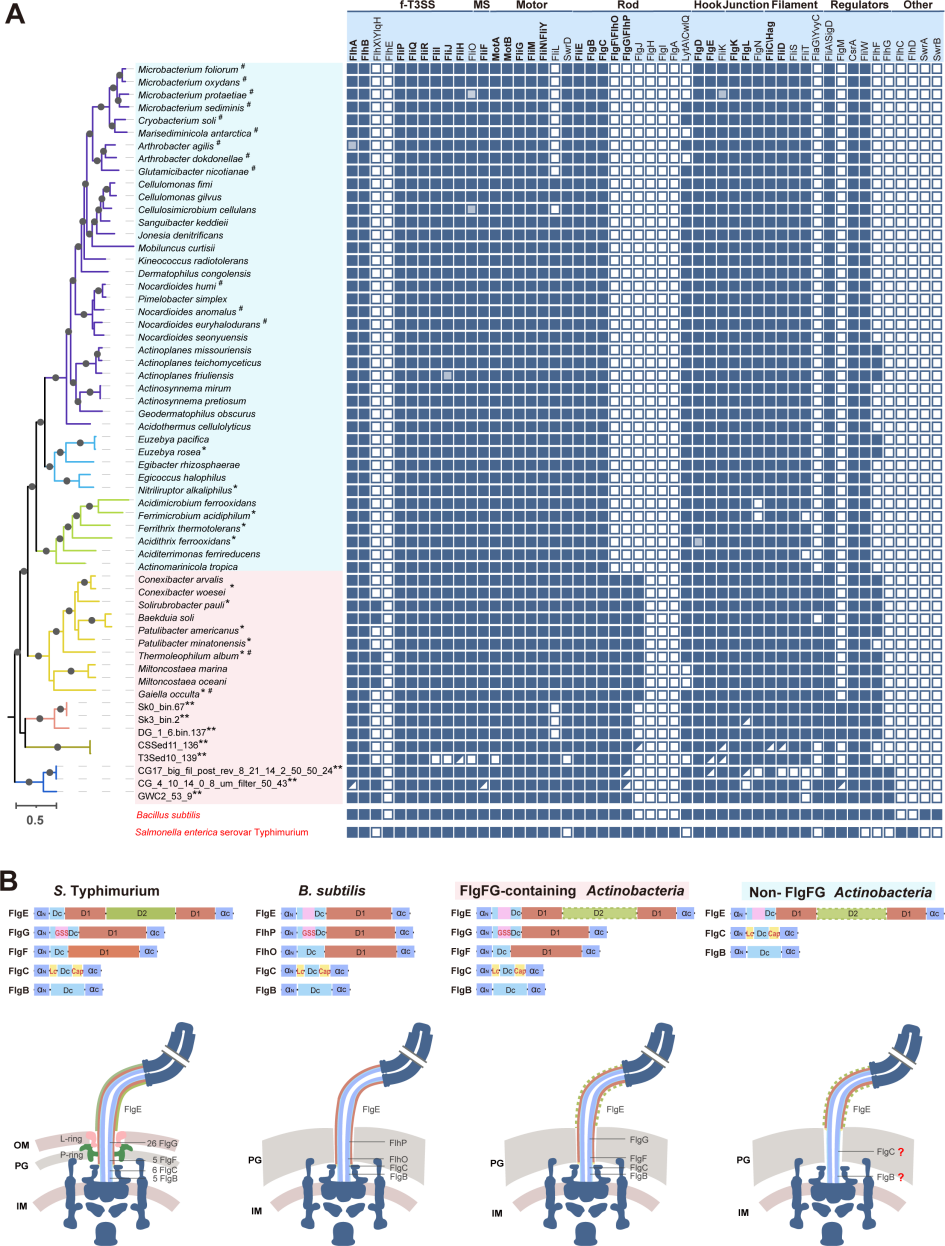
The degenerative evolution of flagella in *Actinobacteria*. (**A**) Flagellar composition in flagellated species of *Actinobacteria*. Left is a species tree taken from Fig. 2C and the branch colors are the same as in Fig. 2C. Actinobacterial species with FlgFG are shaded in a pink box and species without FlgFG are shaded in a light blue box. Species marked with * are incomplete genome assemblies and ** are MAGs; species marked with # represent the absence of a chemosensory system. Right is the presence or absence of 50 flagellar genes in each genome. Solid square represents the presence of the gene; hollow square denotes the absence of the gene; shaded square indicates pseudogene; half square represents the incomplete coding sequence for a protein, mostly found at the edge of the contigs from MAGs thus likely a complete ORF in the genome. In the top line, 24 core genes for bacterial flagella as suggested by Liu and Ochman in reference 36 are highlighted in bold. (**B**) Comparison of the domain organization of FlgB/C/F/G/E in actinobacterial species, diderm model *S*. Typhimurium, and monoderm model *B. subtilis*. Dashed lines of the D2 domain in FlgE indicate that the D2 domain is missing in FlgE homologs of some actinobacterial species. The pink box in FlgE represents the presence of a FlgG-GSS motif like sequence in FlgE. The schematic diagram of flagellar structures below is colored according to the domain colors above. “?” denotes the subunit number of FlgB or FlgC is unknown.

We mapped the presence or absence of all identified flagellar components into the species tree of flagellated actinobacterial species (Fig. 3A). Meanwhile, their compositions were compared with closely related Gram-positive model *Bacillus subtilis* and the simplest Gram-negative model *S*. Typhimurium to infer the characteristics of actinobacterial flagella (30, 31). A prominent feature of flagellar composition in *Actinobacteria* is the presence of FlgF, FlgG [named as FlhO, FlhP in *B. subtilis* (32)] and FlgJ in basal classes but absent in three later classes (Fig. 3A). In both *B. subtilis* and Gram-negative models, the flagellar rod is made up of subunits of FliE, FlgB, FlgC, FlgF, and FlgG that are packed sequentially to transmit basal body rotation to the hook and filament; while the rod cap FlgJ, missing in *B. subtilis* but present in Gram-negative bacteria, acts as a chaperone to usher rod protein polymerization (32, 33). Recent state-of-the-art Cryo-EM studies revealed that in the order of FlgB/C/F/G, these rod components gradually gained extra domains or motifs to increase rod rigidity and ensure adaptation with the hook protein FlgE for torque transmission (34, 35). In *S*. Typhimurium, the proximal rod is made of 5 FlgB and 6 FlgC, constituting a relatively short and single-layered tube; while the distal rod is composed of 5 FlgF and 24 FlgG that form a long and double-layered tube (34, 35). The absence of FlgFG and FlgJ in later classes suggests that the structure and assembly of flagellar rods in these actinobacteria is different from the *S*. Typhimurium model (Fig. 3B).

In order to explore the molecular basis of rod-hook connection without FlgFG, domain analyses, and structure predictions were performed for rod and hook proteins from actinobacterial species. Both FlgB and FlgC homologs in actinobacteria only contain the highly conserved polymerization core D0 (α_N_+α_C_) and Dc domain, and the presence of Lc and Cap region in FlgC but not in FlgB could enable reinforced interaction of FlgC with FlgE (Fig. 3B; Fig. S4A) (34). Hence, it is more likely for FlgC to directly interact with FlgE and the FlgC subunit number should not be limited to six considering the adaptation with FlgE and the thickness of the peptidoglycan layer (Fig. 3B). However, without the D1 domain, neither FlgB nor FlgC can form a more reinforced interaction with FlgE as FlgG does, thus no double-layered tube can be assembled in later classes of actinobacteria without FlgFG (Fig. 3B; Fig. S4A). Next, the structures of actinobacterial FlgE homologs were also predicted and compared with known structures. Interestingly, both sequence alignment and structural comparison showed that in the highly conserved Dc domain, FlgE homologs from actinobacterial species and *B. subtilis* have an extra motif, which is missing in *S*. Typhimurium and some but not all Gram-negative bacteria (Fig. S4C and S5). Of note, the region of this extra motif in actinobacterial FlgE is exactly the unique FlgG-specific sequence (GSS) region in *S*. Typhimurium FlgG (Fig. S4B). The FlgG GSS region is involved in the intersubunit interactions in the distal rod and the maintenance of its rigidity (34, 37). Hence, the presence of a GSS-like motif in the FlgE of actinobacteria and *B. subtilis* is worth structural and functional investigation, which might uncover how a simpler rod can interlock with the hook and rotate.

Surrounding the rod, all actinobacterial species lack FlgH, FlgI, and FlgA that form a bushing complex (LP-ring) through the outer membrane in Gram-negative bacteria, more precisely diderm bacteria (Fig. 3A and B). This result supports the fact that all identified actinobacterial species are monoderm except the mycolic acid-containing *Corynebacteriales*. Hence, we speculate that the last common ancestor of *Actinobacteria* already lost the outer membrane, different from the phylum *Firmicutes* whose common ancestor is diderm and the outer membrane was lost multiple times independently in this phylum as recently proposed (38, 39). Besides, there might be variations in the cell envelop structure between the early FlgFG-containing classes and later non-FlgFG classes since changes in the rod are likely related to its surrounding cell envelop (Fig. 3B). Moreover, non-FlgFG classes also lack other components such as FlhX, FlaG, FlhF, and FlgM, suggesting a simpler flagellar composition en bloc in these later classes, compared to the FlgFG-containing basal classes (Fig. 3A).

Overall, our results showed that flagellar machinery underwent a degenerative evolution in *Actinobacteria*. The early branching classes share more structural components with other bacteria, while the later classes have the simplest rod composition known so far.

### The switch of chemosensory class from F1 to F5

**Fig 4:**
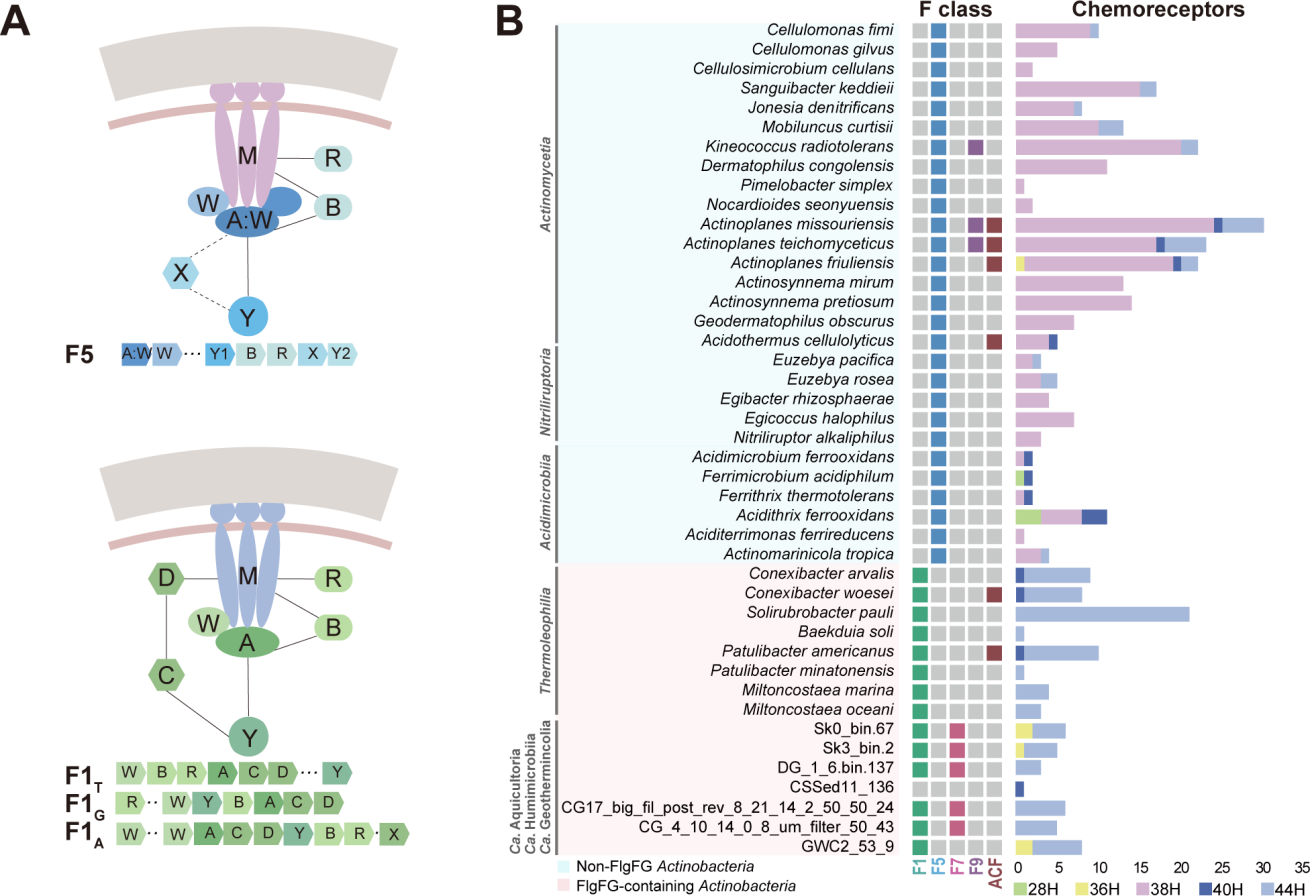
The chemosensory class switch accompanied by flagellar changes. (**A**) Models of F1 and F5 chemosensory classes on the basis of protein composition, gene order, and chemoreceptor type. Abbreviations: A, CheA; W, CheW; A:W, CheA with additional CheW domain; M, chemoreceptor; B, CheB; R, CheR; C, CheC; D, CheD; Y, CheY; X, CheX. For the F1 class, there is no conserved gene order reported (32), and the F1 gene orders in classes *Thermoleophilia*, *Ca*. Geothermincolia, and *Ca*. Aquicultoria are depicted as F1_T_, F1_G_, and F1_A_. (**B**) All chemosensory classes identified in actinobacterial species with chemoreceptor numbers and types shown on the right. The color codes of chemosensory classes and chemoreceptor types are given at the bottom.

Next, we sought to investigate the characteristics of the chemosensory system that is the navigator for flagellar motility in actinobacteria. To facilitate the analyses, we adopted a phylogenomic classification scheme for the chemosensory system defined by Wuichet and Zhulin (40). Interestingly, almost all species from FlgFG-containing classes have F1 chemosensory class, which is featured by the presence of two auxiliary components CheC and CheD (Fig. 4A and B; Table S3). Differently, most species of non-FlgFG classes have an F5 class, distinctive for its conserved gene order (AW...BR) and an additional CheW domain in its kinase CheA (40). Consistently, F1 class containing species mainly have 44H chemoreceptors, which are the cognate type for the F1 class; while species with F5 class mostly have 38H chemoreceptors matching the F5 class (Fig. 4A and B; Table S4). In addition to these two major F classes, some species also have F7, F9, or alternative cellular function (ACF) classes in their genome. For example, the F7 class is only and frequently found in species of the basal classes *Ca*. Aquicultoria and *Ca*. Geothermincolia, and its absence in the class *Ca*. Humimicrobiia might be due to the incompleteness of currently available MAGs of this class (Fig. 4B). F9 class is present in three *Actinomycetia* species and all of these species can form zoospores. Lastly, the ACF class with alternative cellular functions other than flagellar motility is found in six species, three of which belong to the genus *Actinoplanes* with zoospores.

Recent studies revealed that different F classes can form independent chemosensory arrays in a bacterial cell and chemoreceptors of an H category tend to feed into the same array formed by its cognate F class (41–43). Compared to the abundance of F1-specific 44H in basal classes and F5-specific 38H in later classes, the number of chemoreceptors compatible with F7, F9, or ACF classes (36H for F7, 44H for F9, 40H for ACF) are very few (Fig. 4B; Table S4). Based on the sensory capability, it is likely that the F1 class forms the major chemosensory array to direct flagellar motility in basal classes, while the F5 class is the dominant signaling array in later classes. In addition, the clear phylogenetic division of the F1 class and F5 class matches the presence and absence of FlgFG in actinobacteria, suggesting an evolutionary link between the chemosensory class switch and flagellar rod variation. Finally, among the 69 flagellated species, 14 species mostly from several genera of *Actinomycetia* were found to lack any chemosensory system (Fig. 3A). Bacterial species with complete flagellar gene set but no chemosensory signaling pathway are very rare and the first report was *Aquifex aeolicus* when its genome sequence became available (44). However, it remains unclear how a motile bacterium controls its direction without a chemosensory system and our analyses here provided additional actinobacterial species for further behavior studies.

### The upsurge of c-di-GMP enzymes in flagellated species particularly zoospore formers

Flagellar motility can be controlled by secondary messenger c-di-GMP in many ways, such as motor inhibition (45–47), motor switching (48, 49), or transcriptional regulation (50, 51), all of which is achieved by binding to various receptors. Due to the complexity of multilayer regulation and lack of knowledge of all the receptors, the influence of c-di-GMP on flagellar motility cannot be qualitatively and quantitatively determined by genome analysis (52). However, estimation on the c-di-GMP mediated signaling network at the phylum level for *Actinobacteria* will be useful since all studies on c-di-GMP signaling pathways within this phylum focused on non-motile *Streptomyces* and *Mycobacterium* species (53–56).

**Fig 5:**
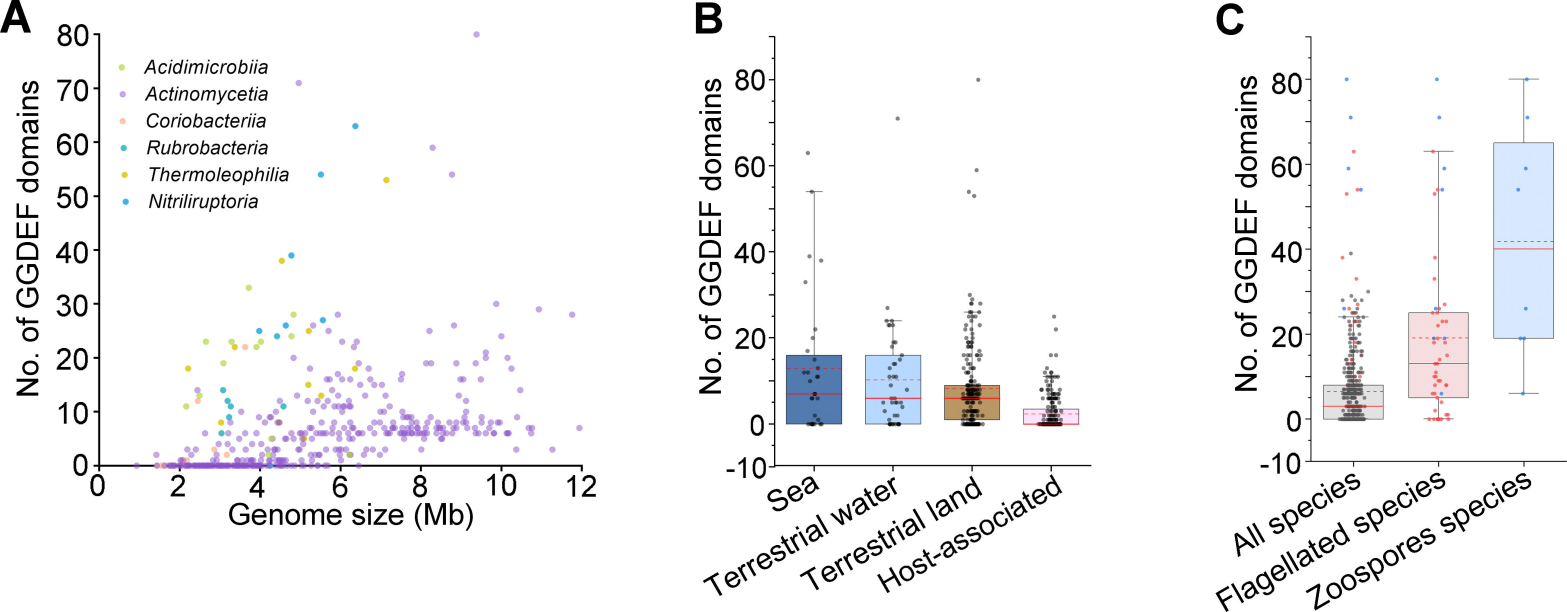
The increase of c-di-GMP enzymes in flagellated species of *Actinobacteria*. (**A**) Relationship between the number of GGDEF domain-containing proteins and the genome size. (**B**) Relationship between the number of GGDEF domain-containing proteins and ecological types. The red solid and dashed lines represent the median and mean, respectively. (**C**) Number of GGDEF domain-containing proteins in non-flagellated species (gray dots), flagellated species (red dots), and zoospore-forming species (blue dots).

In order to roughly assess the size of the c-di-GMP mediated signaling network, the genes that encode turnover enzymes for this molecule were counted for each actinobacterial genome, particularly the signature “GGDEF domain” in charge of c-di-GMP production. The proteins containing GGDEF domain from actinobacterial species varied greatly, from 0 to 80, suggesting that the importance of c-di-GMP signaling pathways can be trivial or oppositely enormous among species of this phylum (https://github.com/Gaolab203/Actinobacteria_flagellar_evolution, S13 Data). Actinobacterial species are well known for their wide range of genome size, from 0.9 Mb to 12 Mb, but the biased abundance of GGDEF domain in these species is not related to the genome size (Fig. 5A). When the species were divided into groups based on their ecological distribution, it is clear that host-associated species generally have less GGDEF domain-containing proteins than free-living groups, but there are no significant differences among species inhabiting the sea, terrestrial water, or soil (Fig. 5B). Then, flagellated species were analyzed separately and they showed significantly higher number of GGDEF domain-containing proteins than the average of all actinobacterial species (Fig. 5C). We noticed that the species with the highest number of GGDEF domains are from the zoospore-forming genus *Actinoplanes* and the oddly high number of c-di-GMP enzymes in *A. missouriensis* has also been pointed out recently (20). Thus, we further analyzed all the actinobacterial species that were reported to form zoospores and they do constitute the portion of species with the most GGDEF domains (Fig. 5C).

The turn-on and turn-off of flagellar genes in zoospore-forming species must be precisely regulated by signal transduction pathways since their motile period is limited to 2–3 h before zoospores germinate or die (23, 24). Thus, the high abundance of c-di-GMP enzymes likely correlates with the control of flagellar gene expression and motility behavior. Meanwhile, the chemosensory system of several zoospore-forming species has extra F9 and/or ACF classes in addition to the F5 class (Fig. 4B), which might also be related to the stringent regulation of flagellar motility and cell cycles.

## DISCUSSION

**Fig 6:**
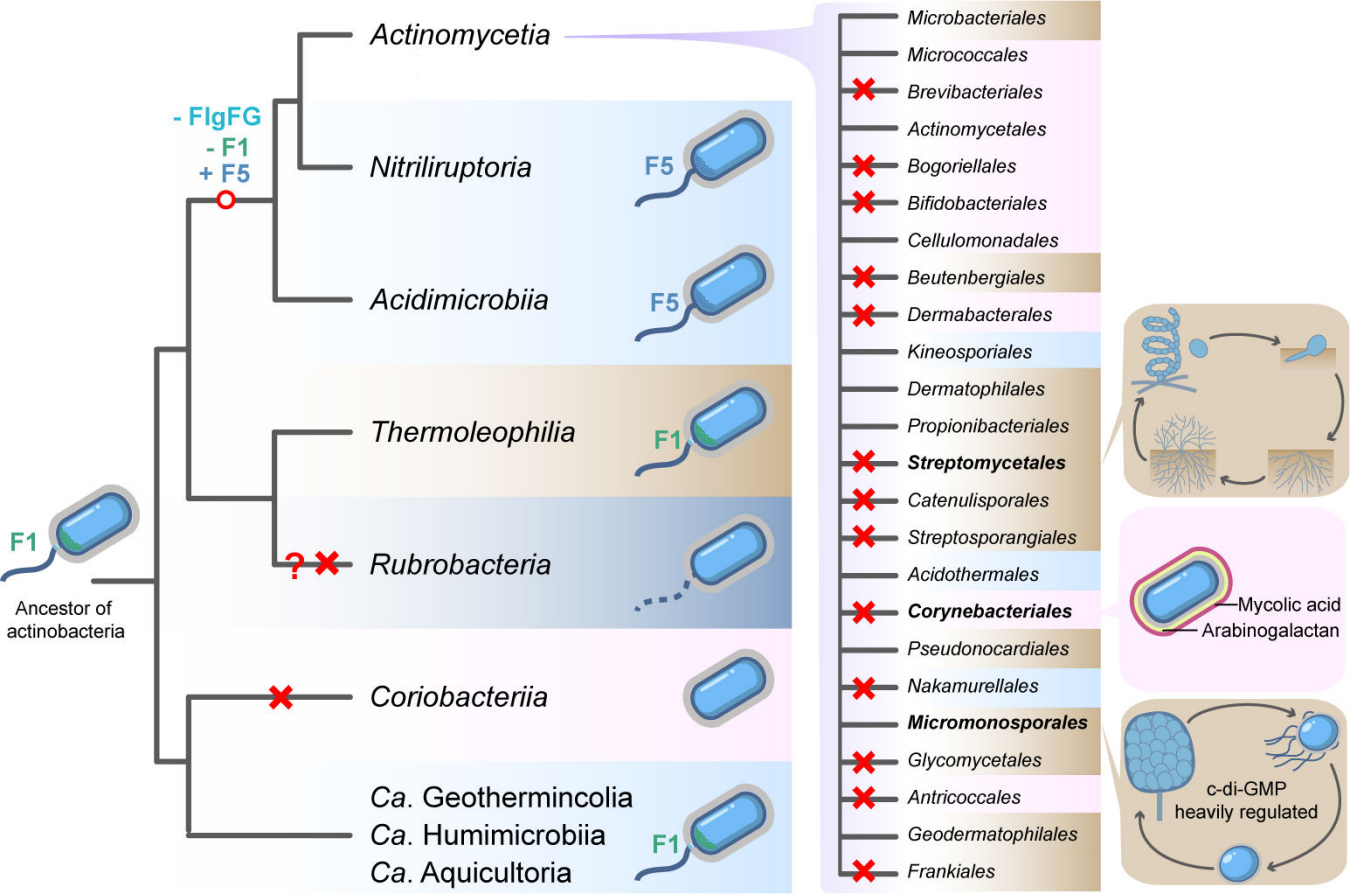
Schematic representation for the evolution of flagella in *Actinobacteria*. In this model, the last common ancestor of all actinobacteria is a flagellated monoderm cell with F1 chemosensory class. Later, when the lineage composed of *Acidimicrobiia*, *Nitriliruptoria*, and *Actinomycetia* diverged, their common ancestor lost distal rod components FlgFG and F1 class but gained another chemosensory class F5. Red cross suggests flagellar gene loss event in the ancestor of each class or each order within the class *Actinomycetia*, and flagellar losses that happen after the lineage diverged are not indicated here. “?” denotes that the flagellar gene loss event for the branch awaits verification from more specie sampling. The shading color of the taxon depicts the ecotype of most of its species, including terrestrial land (drab), terrestrial water (light blue), sea (dark blue), and host-associated (pink). On the right, orders of *Actinomycetia* with unique morphology or life cycle are depicted.

Here, we performed detailed analyses of flagellar evolution in *Actinobacteria*. Our results suggest that the last common ancestor of actinobacteria had a full set of flagellar genes and these genes were vertically passed on to its descendants. As illustrated in a summary model, early branched lineages composed of unicellular species in aquatic environments mostly preserve the flagellar organelle for swimming, while later derived lineages that are host-associated or soil-dwelling plus filamentous tend to lose flagella (Fig. 6).

The role of flagellar motility in bacterial niche adaptation is well known, but the benefits of flagellar loss have been much less studied. Two advantages of flagellar loss can be summarized from previous studies: evasion of host immune surveillance towards flagellin, and energy cost in species with streamlined genomes (10, 57). However, these cannot explain why the class *Actinomycetia* with the most species diversity had massive flagellar gene losses from a motile ancestor, in spite of the fact that half species of this class are free-living and with genome size of >5Mb (Fig. 6; Fig. S1). Compared to the other classes in *Actinobacteria*, most species of *Actinomycetia* except the host-associated lineages are living in soil instead of aquatic environments. On top of it, many of these soil-dwelling species have developed complex life cycles and morphology, which is not seen in other actinobacterial classes and the underneath difference is multicellular growth. Cells that grow in chains better abandon single-cell flagellar motility, which will jeopardize the formation and maintenance of the filament (58). Thus, we speculate that flagellar gene loss, if it is not the trigger, is likely be one of the contributing factors in the evolution of multicellularity in actinobacterial species. Similarly, bacterial groups with multicellular lifestyles such as cyanobacteria and myxobacteria do not have flagella, either (58).

However, multicellularity and mycelium growth are not exclusive to species without flagellar genes. Some flagellated species such as *B. subtilis* can transiently enter the multicellular stage by forming biofilm or cell chains, but their flagellar gene expression is repressed at this stage and the matrix-producing cells are non-motile (59, 60). Likewise, the zoospore formers of *Actinomycetia* only utilize flagellar motility during their unicellular spore period and must abandon their flagellar filaments and motility behavior once germination starts. To maintain the dozens of flagellar genes in these genomes, a trade-off might be a large and complex signal transduction network to coordinate the flagellar gene expression with the cell cycle. Consistently, our results showed that zoospore formers have more chemosensory classes and a much larger number of c-di-GMP enzymes than the other actinobacterial species.

In addition to the massive loss of flagellar gene set in later actinobacterial lineages, the flagellar structure also underwent degenerative evolution in species that preserved this nanomachine. The most significant change is the loss of distal rod components FlgFG in later actinobacterial classes, which indicates that the current working model for rod-hook assembly based on *S*. Typhimurium does not apply here. Since the rod is embedded in bacterial cell envelopes and must co-evolve with the surrounding structure, we hypothesize that there might be changes in the cell wall architecture of later actinobacterial classes compared to the basal classes with FlgFG. Interestingly, accompanying these flagellar compositional changes, the chemosensory system also switched from the F1 class to the F5 class in later actinobacterial classes. It’s known that the chemosensory system co-evolves with flagellar machinery and our recent studies on the phylum *Campylobacterota* proposed an evolutionary scenario for chemosensory class switch caused by flagellar alteration (61). However, there is no clue about how this chemosensory class switch happened in actinobacteria.

In summary, our study here provided new insights into actinobacterial evolution from the lens of flagellar machinery, an overlooked aspect before. The findings here also brought interesting questions to explore in the future. For example, how a simple rod can assemble and rotate in thick peptidoglycan layers or zoospore envelopes; whether the flagellar loss in *Corynebacteriales* was due to host association or the appearance of an unusual outer membrane. Finally, as a product of degenerative evolution, the simplest version of flagella in actinobacteria is a possible model for future reconstitution of a minimal flagellar nanomachine in non-flagellated species or synthetic cells.

## MATERIALS AND METHODS

### Genome data source and phylogenetic analyses

Four hundred and eighty representative species with complete genomes in *Actinobacteria* were obtained from the NCBI RefSeq database in August 2022 (62). Another 23 incompletely assembled genomes of representative species from less sequenced classes including *Nitriliruptoria*, *Acidimicrobia*, *Thermoleophilia*, and *Rubrobacteria* were chosen to improve taxonomic diversity. These 23 incomplete genomes were also downloaded from the NCBI RefSeq database (62). In addition, 35 MAGs from three recently identified actinobacterial classes with genome completeness greater than 75% were also analyzed and these genome sequences were kindly provided by the first author of reference (26). All 538 representative species and related genome information were listed in Table S1 and the taxonomy was based on reference (15).

A maximum likelihood (ML) phylogenetic tree of the representative species was constructed using 120 markers from GTDB-Tk (63). Genome quality was evaluated by CheckM (64), and genomes with <80% completeness and >5% contamination were removed, except for flagellated *Euzebya pacifica* and *Miltoncostaea oceani* with contamination of 7.12% and 6.03%, respectively. Finally, 534 genomes from *Actinobacteria* and 21 representatives from closely related *Firmicutes* phylum were retrieved to reconstruct the species tree. The concatenated multiple sequence alignment generated by GTDB-Tk was used to infer an ML tree with IQ-TREE (65). The best-fit model LG+F+R10 was calculated by ModelFinder (66), and bootstrap was estimated by 1,000 replications. The species tree of 58 representative species with flagellar genes in Fig. 2C was rooted with six genomes from *Firmicutes*, and the methods were similar to those described above, using the best fit model LG+F+R6.

### Analyses of flagellar homologs in *Actinobacteria*

To identify flagellar genes in 538 representative genomes, 50 flagellar proteins from *S*. Typhimurium or *B. subtilis* were used as queries for both BLASTP and PSI-BLAST searches (67). The BLAST queries include 11 fT3SS associated proteins (**FlhA**, **FlhB**, FlhX, FlhE, **FliP**, **FliQ**, **FliR**, **FliI**, FliJ, **FliH**, FliO), MS-ring protein (**FliF**), eight motor associated proteins (**MotA**, **MotB**, **FliG**, **FliM**, **FliN**, FliY, FliL, SwrD), 10 rod associated proteins (**FliE**, **FlgB**, **FlgC**, **FlgF**, **FlgG**, FlgH, FlgI, FlgA, FlgJ, CwlQ),11 hook and filament associated proteins (**FlgE**, **FlgD**, FliK, **FlgK**, **FlgL**, FlgN, **FliC**, **FliD**, FliS, FliT, YvyC/FlaG),10 regulators or other proteins (FlhC, FlhD, SwrA, swrB, FliA, FlgM, CsrA, FliW, FlhF, FlhG). From the BLASTP and PSI-BLAST results, candidates with an E-value of less than e^−5^ were further examined by domain organization using the SMART database (68) and structural similarity using HHpred (69). Highly divergent flagellar proteins such as FliJ, FlgJ, FlgN, FliS, and FliT, were also identified by HMMER searches (70) using HMM profiles from Pfam database (71), and manually confirmed by their gene neighborhoods. As for FlgE, FlgF, and FlgG with high sequence similarity at both D0 and Dc domains, the conserved gene order of *flgD-flgE* and *flgF-flgG* was used as a cue to distinguish the BLAST hits. In addition, an unrooted phylogenetic tree of FlgE, FlgF, and FlgG homologs was constructed. In this tree, the identified FlgE, FlgF, and FlgG formed distinctive clusters with validated homologs from model organisms, ensuring our discrimination of these three proteins (Fig. S6). All identified flagellar proteins were summarized in Table S2 and the flagellar genes were mapped on the linearized chromosomes for visualization of their genomic distribution by RIdeogram in Fig. S3 (72).

An earlier study by Liu and Ochman suggested 24 structural genes as an ancient core set for bacterial flagella based on the phylogenetic occurrence and histories of each of these genes (36). The proteins encoded by these 24 core genes were highlighted in bold in the above paragraph and also in the top line of Fig. 3A. Based on this, if all of the 24 core genes are present in an actinobacterial genome allowing for one or two pseudogene cases, the species is considered as with flagellar gene set and flagellated. Since most flagellar genes from MAGs are incomplete, if at least 10 of the 24 core genes are present in an MAG genome, the species is considered as with a flagellar gene set. Besides, for further detailed analyses, only two or three representative MAGs with relatively intact flagellar gene sets (at least 20 of the 24 core genes) were chosen from each newly identified class as shown in Fig. 3A. Notably, the number of flagellar genes in complete actinobacterial genomes with flagellar gene set ranges from 32 to 41 (Fig. 3A) and based on gene composition and function, all of these species can assemble a functional flagellum.

Twelve flagellar proteins (FlhA, FlhB, FliP, FliQ, FliR, FliI, FliF, FliG, FliM, FliE, FlgB, and FlgC) were selected from 357 bacterial species covering 18 phyla as markers to construct the unrooted flagellar tree. The flagellar proteins chosen for the tree construction have to meet these criteria: universally present in all flagellated species, encoded by the single-copy gene, having a high proportion of alignable positions and readily identified by BLAST search. Individual proteins were aligned by MAFFT using the L-INS-I algorithm, and poorly conserved regions were removed by Gblocks (−b4 = 2, −b5 = *n*) (73, 74). Trimmed alignments were concatenated by PhyloSuite (75), and then used to construct an ML tree by IQ-TREE, using best-fit model LG+F+R10. The flagellar tree in Fig. 2D was rooted with six representatives from *Firmicutes*, and the methods were similar to those described above, using best-fit model LG+F+R6.

The structures of FlgC and FlgE from *B. subtilis*, *A. missouriensis*, and *Conexibacter woesei* in Fig. S4 were predicted by AlphaFold2 (76). The GC contents of the major flagellar gene clusters were calculated by BioEdit (https://thalljiscience.github.io/) (77).

### Analyses of the chemosensory system in *Actinobacteria*

All chemosensory proteins (CheA, CheW, CheB, CheR, CheC, CheD, CheX, and CheX) and chemoreceptors were identified by BLASTP and PSI-BLAST searches. The chemosensory systems were classified based on reference (40), and the H types of chemoreceptors were assigned based on reference (78). The classification of chemosensory proteins was also verified by the MIST database (79). All identified chemosensory proteins and chemoreceptors were summarized in Tables S3 and S4, respectively. The core of a chemosensory system includes four components: chemoreceptor, CheA, CheW, and CheY (80). Hence, if these four components are encoded in an actinobacterial genome, the species is considered with a chemotaxis gene set.

### Analyses of c-di-GMP enzymes in *Actinobacteria*

For the quantification of the c-di-GMP signaling network in actinobacterial species, the GGDEF domain-containing proteins were identified in genomes of all representatives excluding MAGs using HMMER in search of the GGDEF domain (PF00990) (70). All candidate proteins were verified by domain analyses using SMART and HHpred.

## Data Availability

All relevant data are within the paper and its supporting information files. Besides, sequence alignments and tree files for results in Fig. 1B and 2A, C, and D and Fig. S1, S5, and S6 and flagellar protein sequences for MAGs are deposited in Github (https://github.com/Gaolab203/Actinobacteria_flagellar_evolution).

## SUPPLEMENTAL MATERIAL

Fig. S1 mbio.02526-23-s0001.tif Phylogenetic distribution of flagellated species across *Actinobacteria*.

Fig. S2 mbio.02526-23-s0002.tif Flagellar composition and gene clustering pattern in representative species of each order in *Actinobacteria*.

Fig. S3 mbio.02526-23-s0003.tif Genomic distribution of flagellar genes in actinobacterial representative species with complete genomes.

Fig. S4 mbio.02526-23-s0004.tif Structural comparison of FlgC and FlgE.

Fig. S5 mbio.02526-23-s0005.tif Sequence alignment and domain organization of FlgE.

Fig. S6 mbio.02526-23-s0006.tif Phylogenetic tree of FlgE, FlgF, and FlgG.

Supplemental Legends mbio.02526-23-s0007.docx Legends to supplemental figures and tables.

Supplemental tables mbio.02526-23-s0008.xlsx Tables S1 to S4.

ASM does not own the copyrights to Supplemental Material that may be linked to, or accessed through, an article. The authors have granted ASM a non-exclusive, world-wide license to publish the Supplemental Material files. Please contact the corresponding author directly for reuse.
