# Supplementary figures and images for: The common origin and degenerative evolution of flagella in Actinobacteria
Source: mBio. 2023 Nov 29;14(6):e02526-23. doi: 10.1128/mbio.02526-23 (PMC10746217; doi:10.1128/mbio.02526-23)

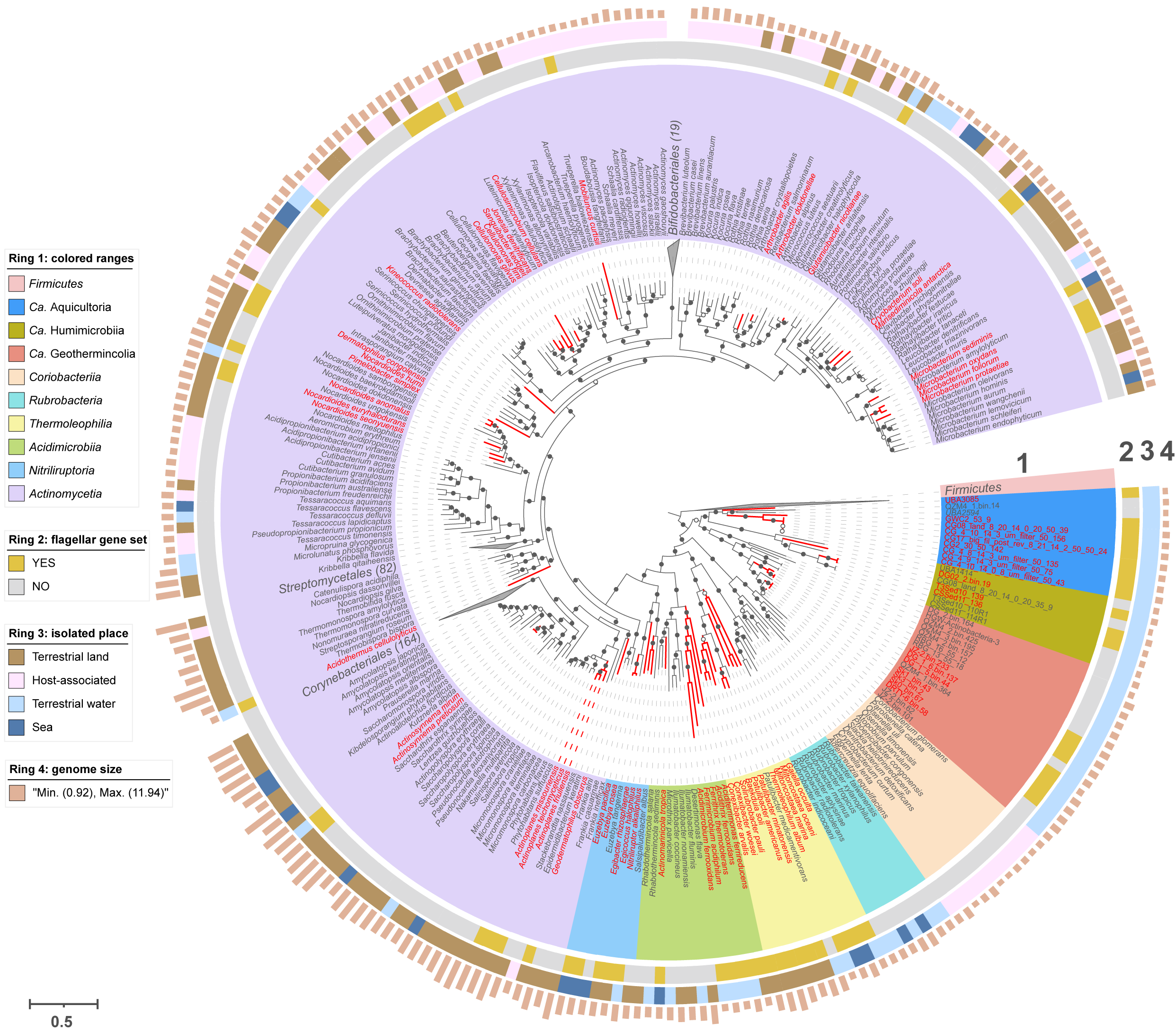

Supplement: Fig. S1 — Phylogenetic distribution of flagellated species across Actinobacteria. [file mbio.02526-23-s0001.tif]

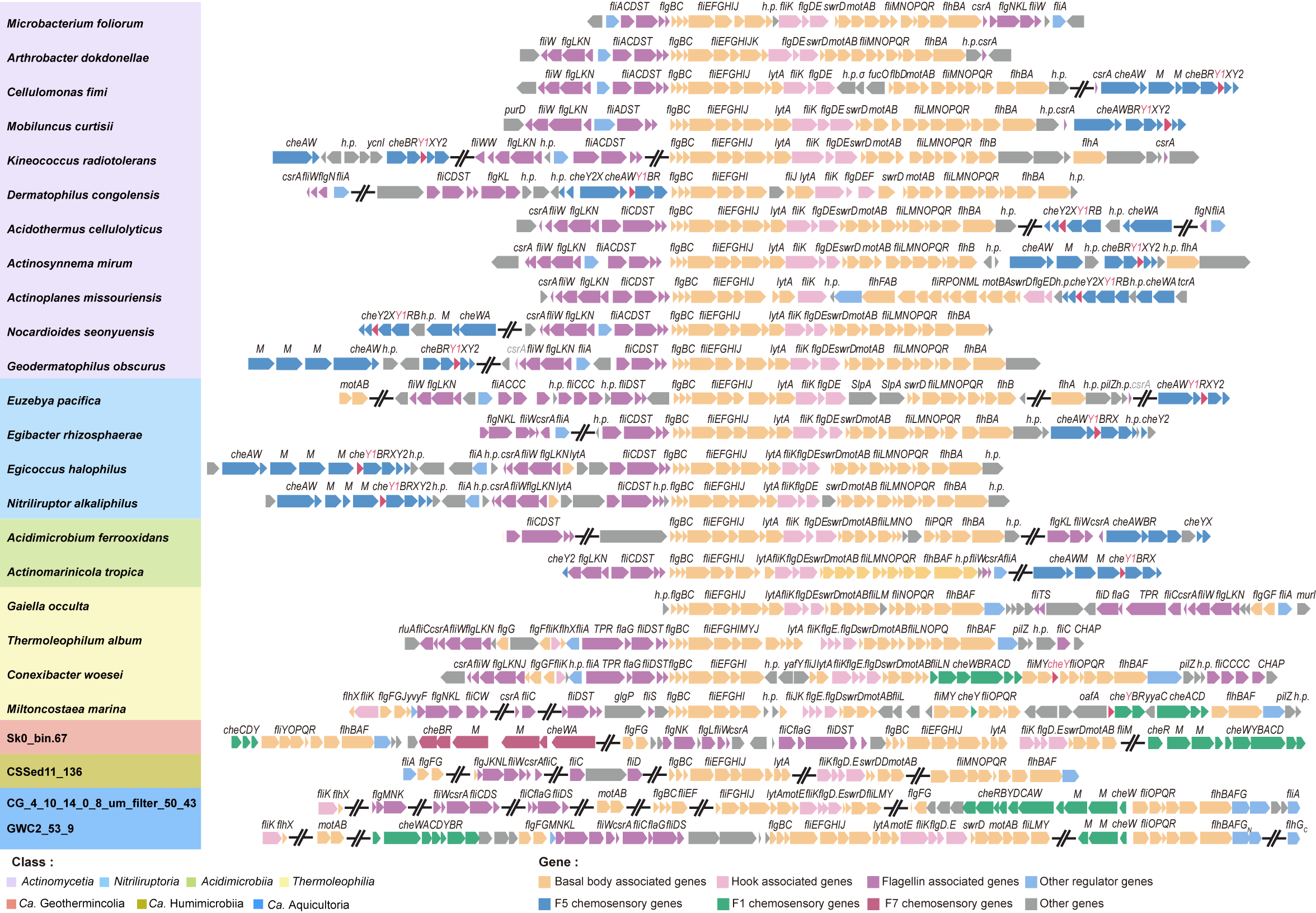

Supplement: Fig. S2 — Flagellar composition and gene clustering pattern in representative species of each order in Actinobacteria. [file mbio.02526-23-s0002.tif]

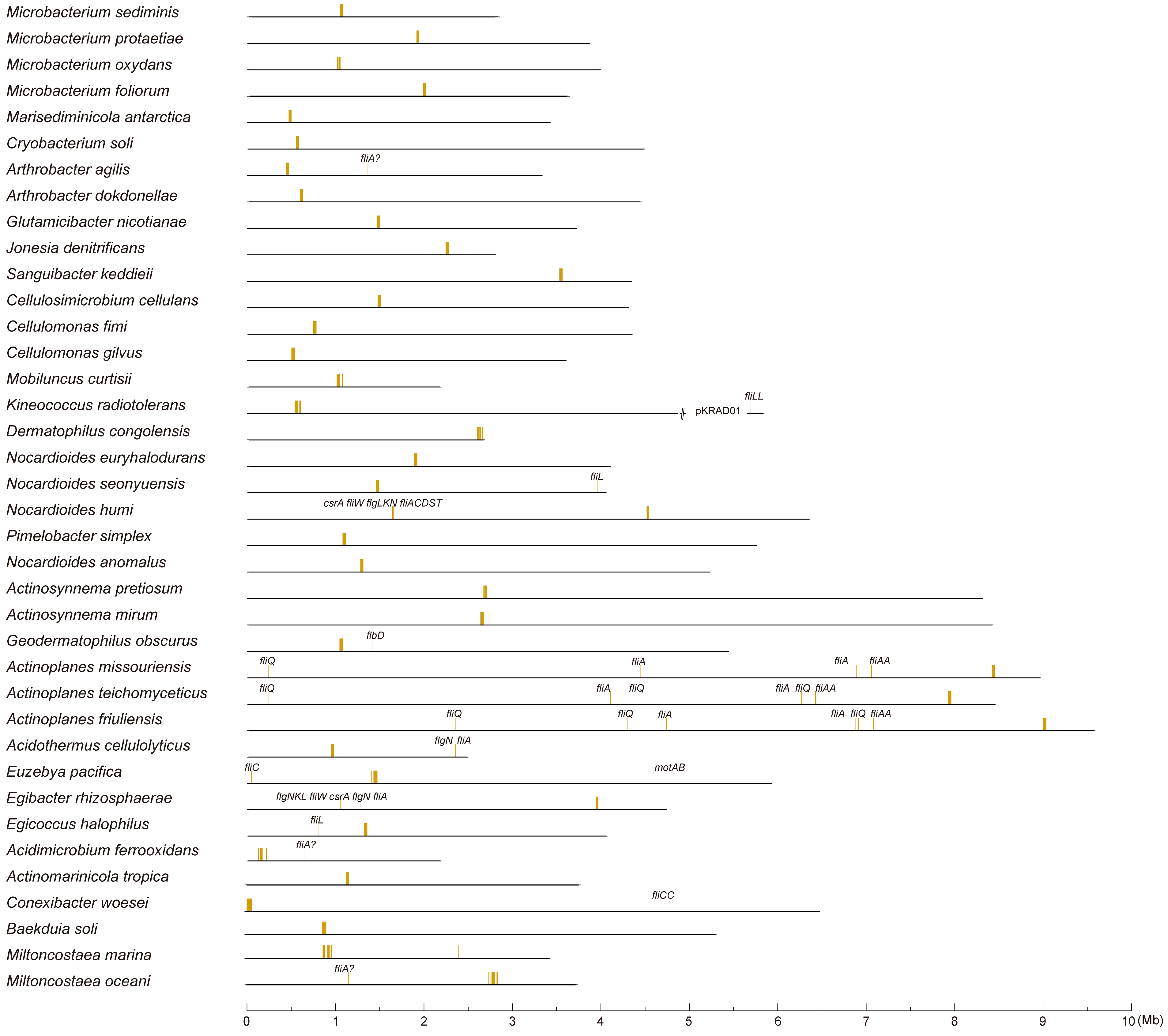

Supplement: Fig. S3 — Genomic distribution of flagellar genes in actinobacterial representative species with complete genomes. [file mbio.02526-23-s0003.tif]

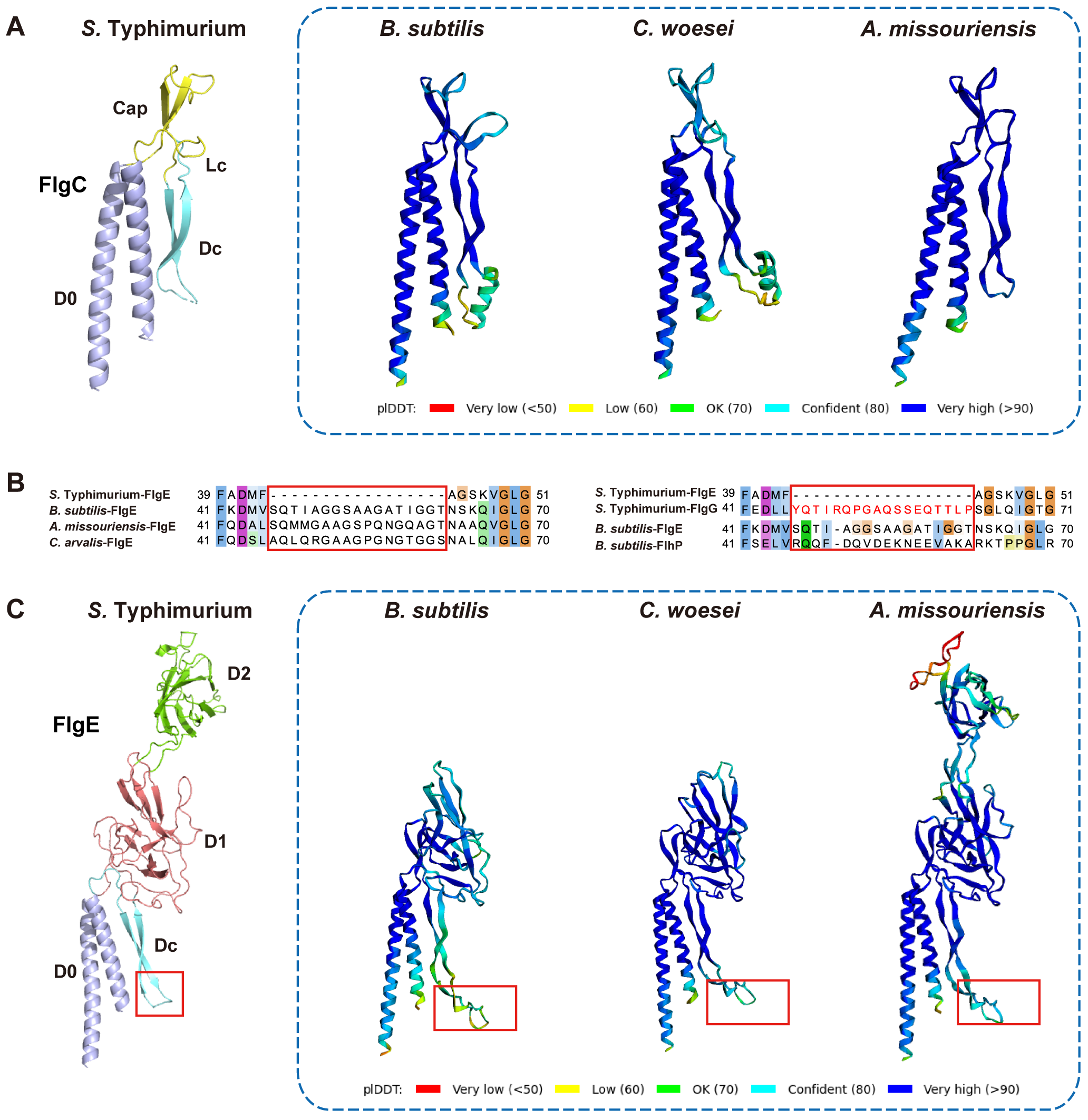

Supplement: Fig. S4 — Structural comparison of FlgC and FlgE. [file mbio.02526-23-s0004.tif]

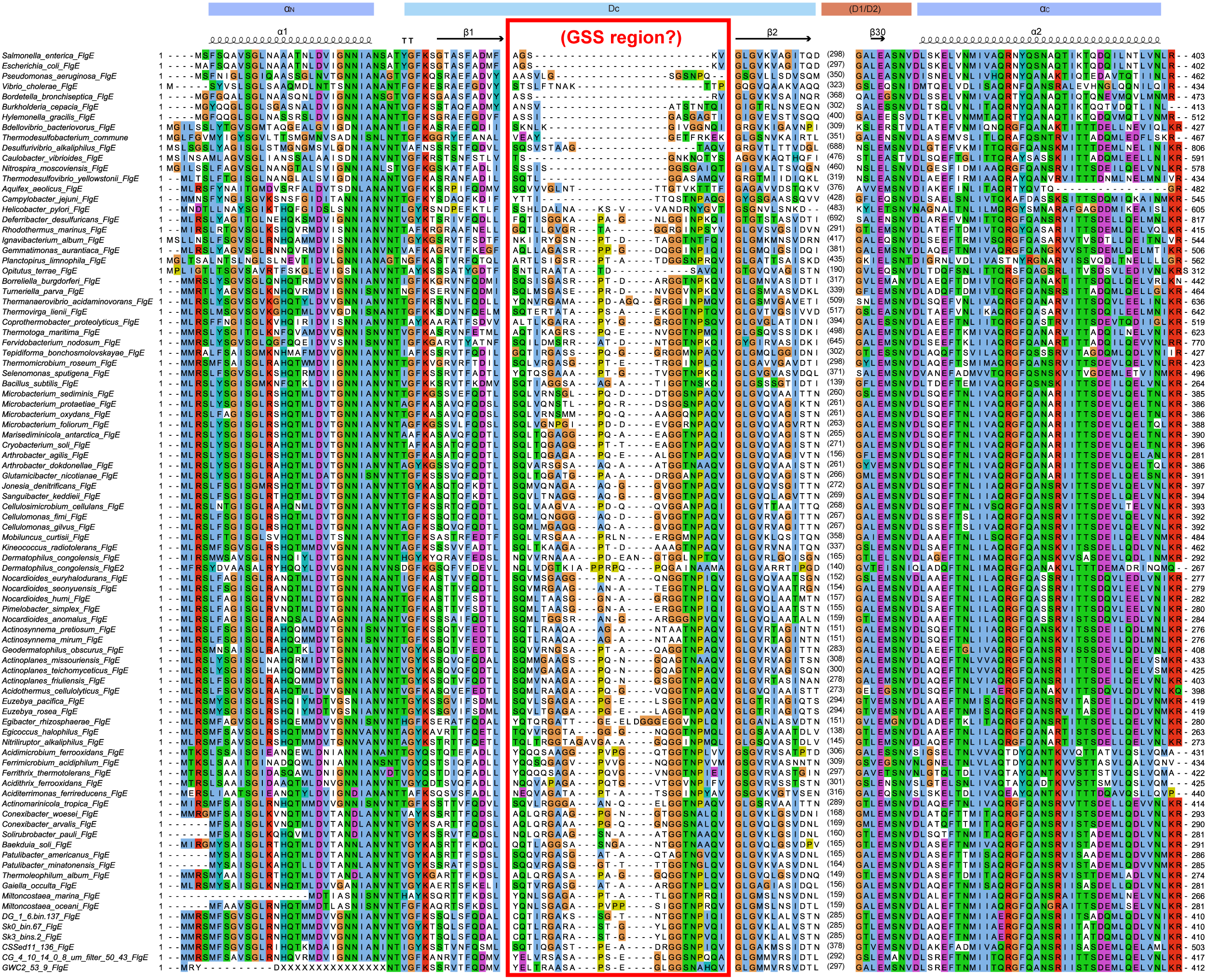

Supplement: Fig. S5 — Sequence alignment and domain organization of FlgE. [file mbio.02526-23-s0005.tif]

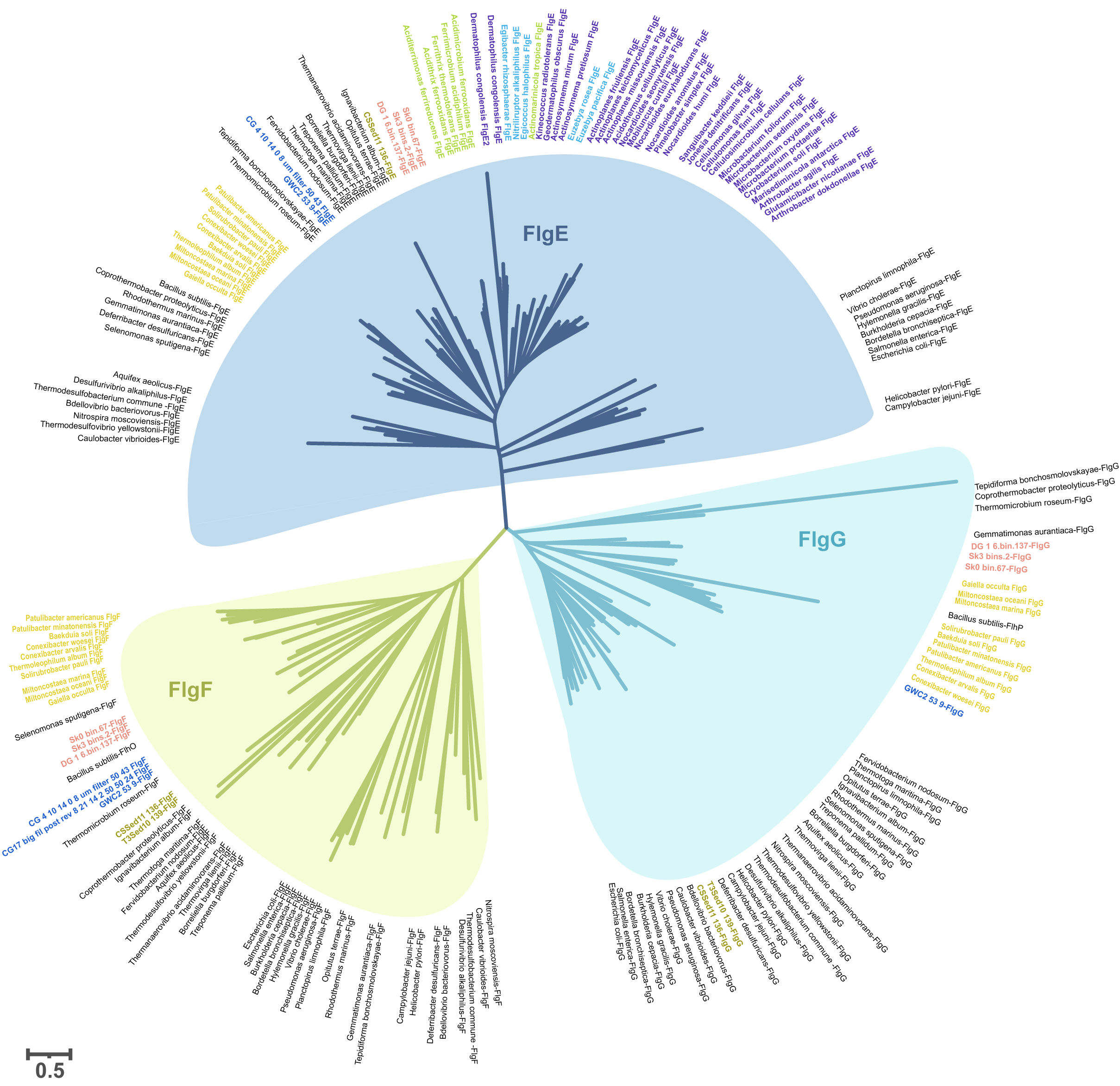

Supplement: Fig. S6 — Phylogenetic tree of FlgE, FlgF, and FlgG. [file mbio.02526-23-s0006.tif]
