# Supplementary material for: The common origin and degenerative evolution of flagella in Actinobacteria
Source: mBio. 2023 Nov 29;14(6):e02526-23. doi: 10.1128/mbio.02526-23 (PMC10746217; doi:10.1128/mbio.02526-23)
Supplement: Supplemental Legends — Legends to supplemental figures and tables. [file mbio.02526-23-s0007.docx]

**Supporting information:**

**S1 Fig**. Phylogenetic distribution of flagellated species across *Actinobacteria*. A maximum likelihood phylogenetic tree of *Actinobacteria* based on a concatenated alignment of 120 marker proteins generated by GTDB-Tk is shown in the middle. Circles above each node represent bootstrap values (n = 1000 replicates) of ≥ 90% (solid) or ≥ 70% (hollow). The color range in ring 1 underneath species names is based on the taxonomic rank class (see taxonomy legend on the top left); ring 2 shows the sporadic distribution of flagellated species, and correspondingly the branches and strain names with flagellar genes are highlighted in red; ring 3 shows the isolated place of each strain; each bar of the outmost ring 4 corresponds to the genome size. Three order *Corynebacteriales*，*Streptomycetales*，and *Bifidobacteriales* from the class *Actinomycetia* without any flagellated species are collapsed as triangle to save space. The zoospore-forming *Actinoplanes* species are highlighted by red dashed lines.

**S2 Fig**. Flagellar composition and gene clustering pattern in representative species of each order in *Actinobacteria*.

**S3 Fig**. Genomic distribution of flagellar genes in actinobacterial representative species with complete genomes. All actinobacterial genomes are linearized here and the flagellar genes represented by ochre strips are generally clustered in a single genomic locus. Some scattered flagellar genes are labeled above the linearized genomes.

**S4 Fig**. Structural comparison of FlgC and FlgE. **(A)** Structural comparison of FlgC. Left, FlgC from *S.* Typhimurium (PDB ID: 7E82); right, the structures of FlgC from *B. subtilis*, *Conexibacter woesei*, and *Actinoplanes missouriensis* predicted by AlphaFold2. The prediction accuracy of per-residue is illustrated by pLDDT. **(B)** Sequence alignment of N-terminal region of FlgE and FlgG. The FlgG-specific sequence (GSS region) is highlighted in red, and the corresponding region of FlgE is indicated by red boxes in both (B) and (C). **(C)** Structural comparison of FlgE from four species. The fragment in FlgE corresponding to GSS region from FlgG are indicated in red box.

**S5 Fig**. Sequence alignment and domain organization of FlgE. The sequences corresponding to GSS region from FlgG are indicated in red box.

**S6 Fig**. Phylogenetic tree of FlgE, FlgF and FlgG. FlgE (dark blue), FlgF (pale green) and FlgG (turquoise) from diverse phyla are clustered as monophyletic groups, respectively. The representative species of *Actinobacteria* are highlighted according to their belonging classes: *Actinomycetia* (purple), *Nitriliruptoria* (Cyan), *Acidimicrobia* (green), *Thermoleophilia* (yellow), *Ca.* Geothermincolia (red), *Ca.* Humimicrobiia (olivine), *Ca*. Aquicultoria (blue).

**S1 Table**. Summary of analyzed species and their characteristics.

**S2 Table**. Summary of flagellar composition in actinobacterial genomes.

**S3 Table**. Summary of chemosensory components in actinobacterial genomes.

**S4 Table**. Summary of chemoreceptor types in actinobacterial genomes.
